# Supplementary material for: SomaticSignatures: inferring mutational signatures from single-nucleotide variants
Source: Bioinformatics. 2015 Jul 10;31(22):3673–5. doi: 10.1093/bioinformatics/btv408 (PMC4817139; doi:10.1093/bioinformatics/btv408)
Supplement: Supplementary Data [file supp_31_22_3673__index.html]

SomaticSignatures: inferring mutational signatures from single-nucleotide variants — SomaticSignatures: inferring mutational signatures from single-nucleotide variants — SomaticSignatures: inferring mutational signatures from single-nucleotide variants — Supplementary Data 

# SomaticSignatures: inferring mutational signatures from single-nucleotide variants

## Supplementary Data

files

- Supplementary Data - pdf file
