# Supplementary material for: SomaticSignatures: inferring mutational signatures from single-nucleotide variants
Source: Bioinformatics. 2015 Jul 10;31(22):3673–5. doi: 10.1093/bioinformatics/btv408 (PMC4817139; doi:10.1093/bioinformatics/btv408)
Supplement: Supplementary Data [file supp_btv408_somatic-signatures-figS1.pdf]

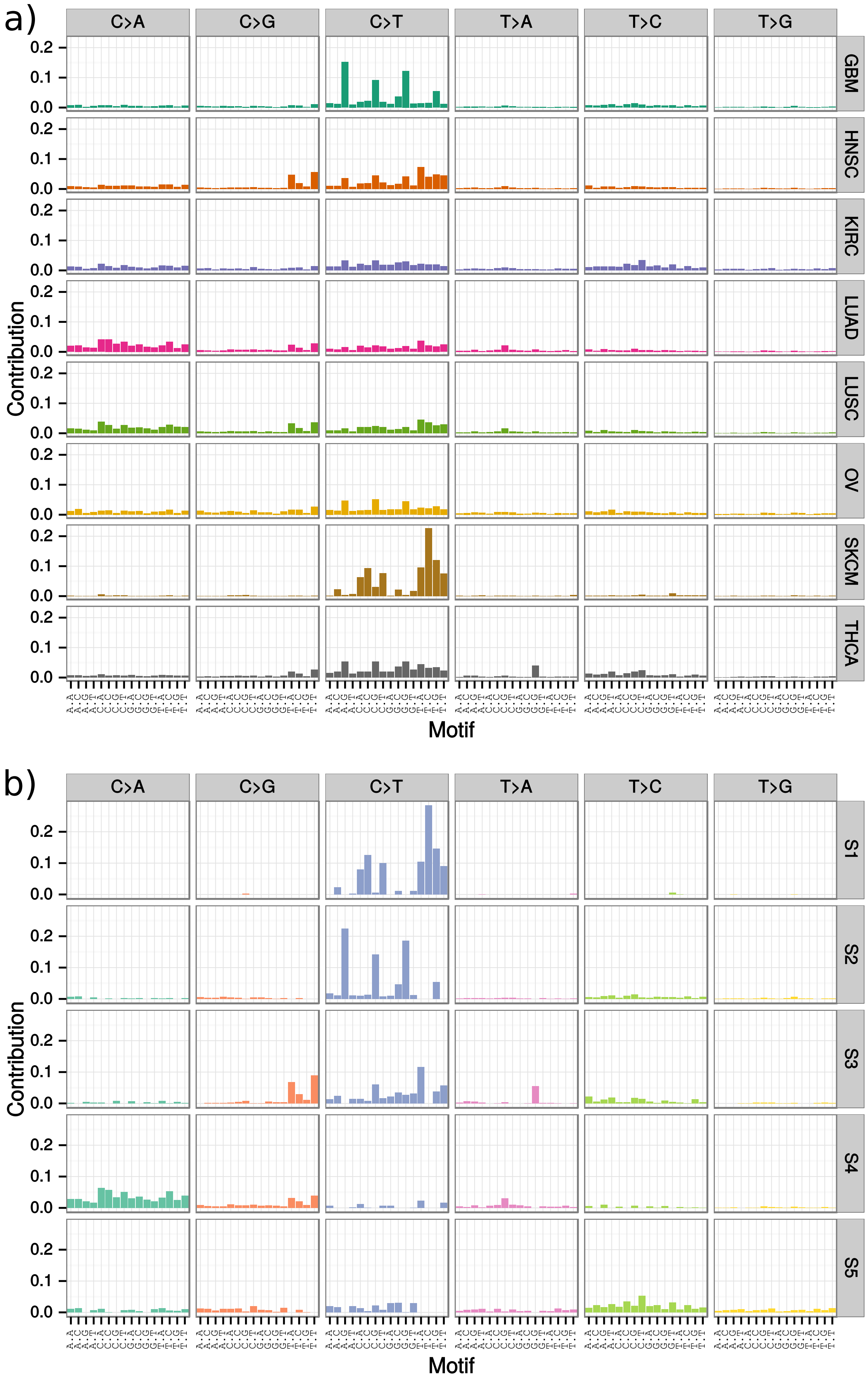

a)
b)
C>A
C>G
C>T
T>A
T>C
T>G
GBM
HNSC
KIRC
LUAD
LUSC
OV
SKCM
THCA
S1
S2
S3
S4
S5
0.0
0.1
0.2
Contribution
Motif

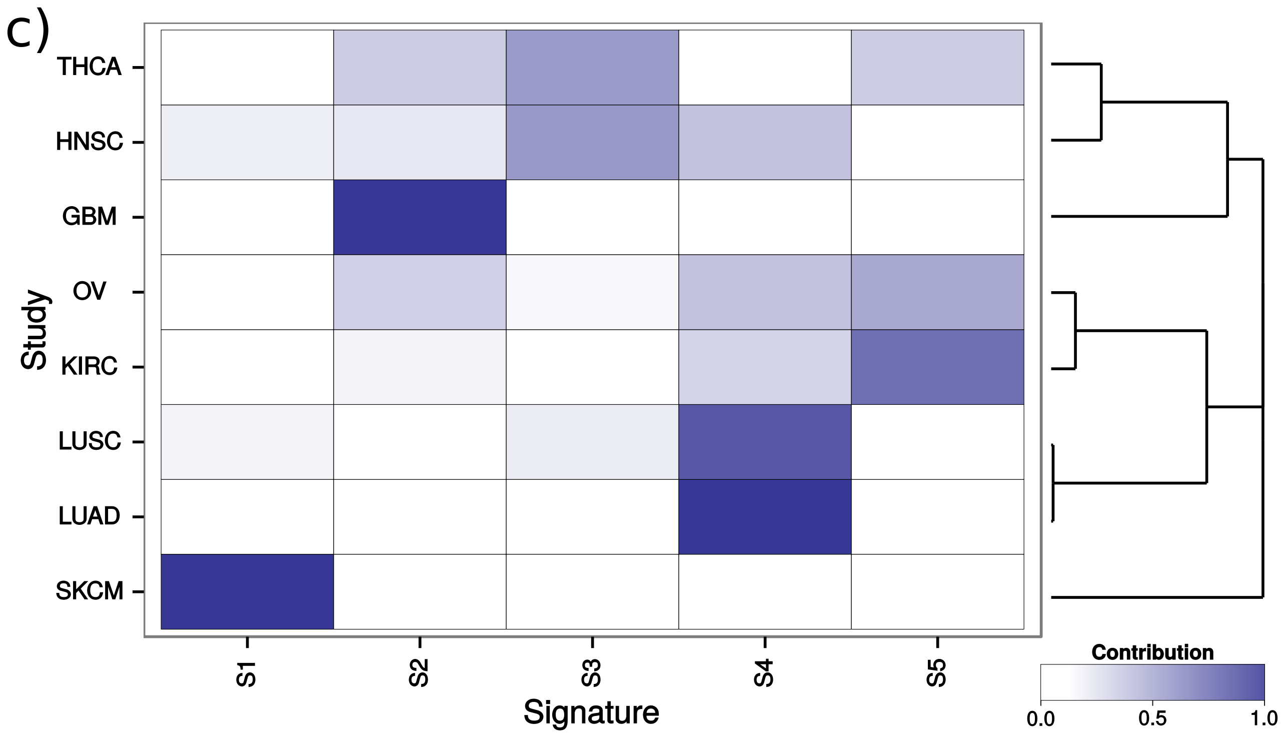

Supplementary Figure S1: Annotated high-resolution version of Figure 1

Analysis of mutational signatures for eight TCGA studies (Gehring, 2014). The observed mutational spectrum of each study (panel a) was decomposed into 5 distinct mutational signatures S1 to S5 (panel b, where the sum of all contributions per sample is normalized to 1) with NMF. The presence of these signatures in the studies (panel c), as shown by hierachical clusting with cosine similarity as distance measure, underlines the similarities in mutational processes of biologically related cancer types.

Cancer types:
- GBM: glioblastoma multiforme
- HNSC: head and neck squamous cell carcinoma
- KIRC: kidney chromophobe
- LUAD: lung adenocarcinoma
- LUSC: lung squamous cell carcinoma
- OV: ovarian serous cystadenocarcinoma
- SKCM: skin cutaneous melanoma
- THCA: thyroid carcinoma
